# The Coming-of-Age Transition Care for Adolescents with Rheumatic Disease—Where Are We and What Have We Done in Asia?

**DOI:** 10.3390/jcm10040821

**Published:** 2021-02-17

**Authors:** Kai Liang Teh, Sook Fun Hoh, Thaschawee Arkachaisri

**Affiliations:** 1 Rheumatology and Immunology Service, Department of Pediatric Subspecialties, KK Women’s and Children’s Hospital, Singapore 229899, Singapore; teh.kai.liang@singhealth.com.sg; 2 Division of Nursing, KK Women’s and Children’s Hospital, Singapore 229899, Singapore; hoh.sook.fun@kkh.com.sg; 3 Pediatric Academic Clinical Program, Duke-NUS Medical School, Singapore 169857, Singapore

**Keywords:** healthcare transition, transitional care, young adults, adolescents, rheumatology, rheumatic disease, review

## Abstract

The transition from pediatric to adult health care is a challenging yet important process in rheumatology as most childhood-onset rheumatic diseases persist into adulthood. Numerous reports on unmet needs as well as evidence of negative impact from poor transition have led to increased efforts to improve transition care, including international guidelines and recommendations. In line with these recommendations, transition programs along with transition readiness assessment tools have been established. Despite these efforts, there are still a lot of work to be done for transition care in rheumatology. This review article focuses on how transition care in rheumatology has developed in recent years and highlights the gaps in current practices.

## 1. Introduction

With progress in medical care, the prognosis of chronic diseases of childhood onset has improved over the years [1]. As more children survive to adulthood, there is also a need to transit them to adult healthcare services. However, the transition from pediatric care to adult care is often a challenging time for adolescents and young adults (AYA) with chronic medical conditions and their healthcare providers [2]. Transition of care is not simply the physical transfer of patients from pediatric to adult-based medical care. Transitional care is defined by the Society for Adolescent Medicine as “the purposeful, planned movement of adolescents and young adults with chronic physical and medical conditions from child-centered to adult-oriented healthcare systems” [3], which includes an entire process of preparing for the transfer, assessing readiness and ensuring its completion.

A study done to explore the attitudes of 138 physicians and allied health professionals attending the Pediatric Rheumatology European Society (PRES) Congress towards transition showed that 87% were supportive of AYA with active rheumatic diseases receiving follow-up care from adult rheumatologists rather than remaining under pediatric care [4]. However, many expressed dissatisfactions with the state of transition processes for childhood-onset rheumatic diseases [5,6]. With that, the importance of transition is increasingly acknowledged and significant progress has been made in the understanding of transition care in rheumatology over the last few decades, including development of more structured transition care programs [7,8,9,10,11]. Studies have also been conducted to evaluate the outcomes of these programs, leading to international guidelines and recommendations based on lessons drawn [12]. This review aims to explore how transition care in rheumatology has developed in recent years and consider the gaps in current practices, with emphasis on unique transition needs in the Asian cultural context.

## 2. Recognizing the Need for Transition in Rheumatology

Transition care is important in rheumatology, since most childhood-onset rheumatic diseases persist into adulthood. More than half of patients with Juvenile Idiopathic Arthritis (JIA) experienced active disease into adulthood and required ongoing management of immunosuppressants [13,14,15]. Childhood-onset Systemic Lupus Erythematosus (SLE) is a lifelong disorder with higher morbidity and mortality compared to adult-onset lupus [16]. In a longitudinal Dutch study, the majority of adults with childhood-onset SLE developed significant end-organ damage at a young age and did not achieve drug-free remission, highlighting the importance of continued care for AYA with childhood-onset SLE even when they reach adulthood [17]. Although the long-term outcome of patients with Juvenile Dermatomyositis (JDM) is not as well established, there are still studies that report ongoing disease activity, impaired physical function and decreased quality of life in adults with JDM [18,19]. In low- and middle-income countries, rheumatic fever remains a serious public health problem that commonly occurs in childhood with life-long disability [20]. Without a planned transition care, there is a risk of worsening disease activity in AYA with rheumatic diseases [21]. In a retrospective, single-center study of 31 patients with chronic rheumatic diseases (52% SLE, 16% mixed connective tissue disease 16% JIA, 13% antiphospholipid antibody syndrome and 3% vasculitis), one-third of patients were hospitalized for disease flare in the year before transfer and another one-third experienced an increase in disease activity in the post transfer year [22]. Lack of proper transition plan will therefore result in greater disease damage, poorer quality of life and greater healthcare cost incurred as these AYA progress into adulthood.

The care of AYA with rheumatic diseases has also changed dramatically over the last few decades with increasing use of biological agents and promise of new targeted therapies [23,24]. Recognizing the need for transition, various recommendations have been published to urge systemic improvement in transition care. The 2014 Institute of Medicine report, “Investing in Health and Well Being of Young Adults,” highlighted the transition from pediatric to adult health care as an important component of improving the health of young adults, particularly those who have chronic disease [25]. A consensus statement issued by American Academy of Pediatrics (AAP), the American Academy of Family Physicians (AAFP) and the American College of Physicians-American Society of Internal Medicine (ACP-ASIM) in 2002 set the goals for all physicians taking care of AYA with special healthcare needs to be equipped with the skills to facilitate the transition process [26].

Despite increasing awareness of the need for transition care, AYA still report unmet needs in this area [27,28]. Many of these unmet needs have not changed from earlier reports, signifying that there is still a discordance between the published recommendations and actual execution of transition plans [29]. A recent study published in 2021 involving 157 AYA with rheumatic diseases reported that two-third of them were not aware about transition care [30]. Another survey of 115 rheumatology centers in 22 European countries reported that only 27% had a formal written transition policy [31]. Majority of North American pediatric rheumatologists still did not consistently address healthcare transition with patients even with growing literature on transition care and improvement in transition barriers [32]. This is in part due to a lack of general consensus on the best practices in transition care.

## 3. Development of Rheumatology Transition Programs

Transition care in pediatric rheumatology can be variable and is often dependent on many factors related to the local healthcare system, culture and healthcare funding. There are relatively few well-described models for transition of pediatric rheumatology patients into adult rheumatology care. Sawyer et al., described three general models for transition care for adolescents with chronic illness: (1) disease-focused transition from pediatric care to an adult subspecialist, (2) primary care–coordinated care spanning adolescent to adult care and (3) generic adolescent health services, with care coordinated by adolescent care providers [33]. A primary care–based model is not usually feasible because of the complexity of rheumatic diseases and well-developed adolescent health services are not available in many centers. Therefore, the most practical model for transition care for AYA with rheumatic diseases is a disease-focused transition from pediatric to adult rheumatology services.

**Figure 1:**
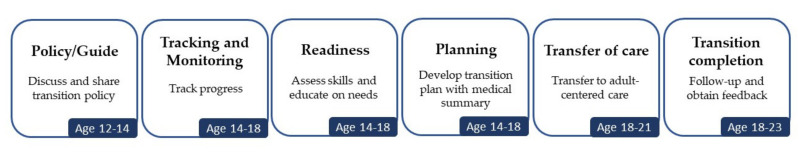
The Six Core Elements by Got Transition which define the basic components of a structured transition process.

Based on the available models of transition care, recommendations have been published to align transition programs in their broad categories of processes to improve overall transition care. In 2011, the AAP, AAFP and American College of Physicians (ACP) jointly outline their suggested approach including the need to assess readiness, patient education, preparation of a medical summary and communication between the pediatric and adult healthcare providers [26]. Got Transition, a federally funded national resource center on healthcare transition in the United States, developed a structured approach called the Six Core Elements of Health Care Transition (Figure 1), which define the basic components of a structured transition process and include customizable tools for each core element [34].

Children with rheumatic disease have a wide range of transition needs based on multiple factors such as disease activity, readiness, cognitive and executive functioning proficiency, home support structures and socioeconomic factors [35]. To assist with implementation of the Six Core Elements, the American College of Rheumatology (ACR) joined the ACP Pediatric to Adult Care Transitions Initiative and formed the ACR Transition Work Group. They developed a subspecialty-specific toolkit tailored to pediatric and adult rheumatologists to assist them in transitioning their patients [36]. In the UK, McDonagh et al., proposed a transition program based on needs assessments using focus groups, a national survey of health professionals, Delphi analysis and retrospective case audits [37]. Tucker and Cabral proposed a transition model involving joint clinic between pediatric and adult rheumatologists, with assistance of a multidisciplinary team [38]. This clinic encouraged patient independence while discouraging over reliance on parents in in making medical management decisions. Rettig and Athreya described a rheumatology specific transition model that uses a multidisciplinary team and a nurse coordinator [39]. The program comprises pre-transitional assessments and interventions that address common adolescent issues.

To integrate the existing models of transition of care for AYA with juvenile onset rheumatic diseases, a task force of the Pediatric Rheumatology European Society (PRES)/European League Against Rheumatism (EULAR) developed the first international set of recommendations and standards. It includes 12 consensus recommendations of “essential/minimal” and “ideal/optional” components of transitional care established via international Delphi analysis and systemic literature review. The recommendations, in agreement with other taskforces [40,41], emphasized the importance of identifying key individuals, the integral role of AYA and their families, written communication, agreed policy, training and clarity of roles within teams [12]. More recently in 2018, a transitional care checklist was developed to facilitate health professionals in providing transition care for AYA and with rheumatic diseases and their families. This checklist achieved strong international consensus and is recommended to be used by physicians taking caring of adolescents aged ≥12 years at times of transition [42].

## 4. Assessing Outcomes from Rheumatology Transition Programs

Although there are multiple international guidelines and recommendations on transition care of AYA with rheumatic diseases, only few established transition programs have been described in literature. A systematic review and critical appraisal of existing transitional care programs in rheumatology identified eight transition programs in six countries [7]. Five other evaluated programs were subsequently reported [43,44,45,46,47]. Common key features include a written transition policy, individualized planning and transition coordinator. However, huge variation in structures, staffing and processes among the programs still exists. Nonetheless, the emergence of transition programs provided valuable insights into the common barriers of transition, important factors in determining readiness and practices that improve success of transition. In an evaluation of a pediatric rheumatology transition clinic in Canada, one of the most important factors identified in determining readiness for transfer was confidence in managing their disease independently [44]. Allowing AYA to assume primary responsibility in managing their disease should therefore be a focus for centers that are looking at refining or developing their transition programs. Tucker and Cabral described a model of a “young adults with rheumatic disease” (YARD) clinic for young people aged 18–24 years involving both pediatric and adult rheumatology professionals to facilitate uninterrupted healthcare provision of age and developmentally appropriate care [38]. This provides a framework for centers which may be looking at setting up similar clinics.

In the implementation of transition programs, various transition readiness measures and checklists have also been developed. Examples include the Transition Readiness Assessment Questionnaire (TRAQ), TR(x)ANSITION Scale and AM I ON TRAC (Taking Responsibility for Adolescent/Adult Care) [48,49,50]. Apart from the Readiness for Adult Care in Rheumatology (RACER) questionnaire, most of these transition readiness measures are not specific to rheumatology [51]. Furthermore, high scores on many of these transition measures do not correlate with successful transition [43], indicating that these transition tools may not have holistically addressed the biopsychosocial aspects of transition but instead focus only on disease and health-related factors. The most robustly validated transition readiness tool to date as evaluated by Zhang et al., is the non-condition specific tool, TRAQ [52]. As there are many transition issues unique to rheumatology including self-administration of subcutaneous medication, body image and psychosocial impact from chronic steroid use as well as effect of Disease Modifying Anti-Rheumatic Drugs (DMARDs) on child bearing, this calls for greater research and development of more robust transition tools that are specific to rheumatology. As most rheumatic diseases can be managed in the clinic setting, there are also additional challenges of assessing transition readiness including limited time, limited personnel and the need to complete other questionnaires as part of disease core set measures. When studying transition readiness, these factors and their impact on any assessment should be considered.

In the systematic review of transition of care programs performed by the European League Against Rheumatism/ Paediatric Rheumatology European Society (EULAR/PRES) working group, an important shortcoming in rheumatology transition programs was also evident—the lack of standardized measures of outcomes and effectiveness. Of the eight transition programs assessed in the systematic review, only the “Growing up and moving on” and “DON’T RETARD” project included outcome measures, both of which showed that the implementation of a transition program can improve health-related quality of life, knowledge and satisfaction in AYA with JIA [53,54]. Objective evaluation of transition programs for AYA with other rheumatic diseases is otherwise not as well published. This is similar to other studies of transition in other chronic diseases, where the need for validated measures of transfer satisfaction is highlighted [55]. There have been several proposed quantifiable outcomes to measure successful transition. A consensus-based proposal regarding outcome indicators for successful healthcare transition was recently made albeit not specific to rheumatology [56]. Developing standardized measures of outcomes and effectiveness of transition programs will be paramount in comparing transition programs and defining best models of transition in rheumatology. In addition to developing outcome measures, it is equally important to develop and establish national programs for rheumatic diseases that are prevalent in individual countries. Longitudinal data from national registries can provide important information on actual transition outcomes of patients which can be used to refine and improve transition programs [57].

## 5. Transition Programs—The Asian Perspective

Much has been discussed about the evolution of transition care for AYA with rheumatic diseases as well as gaps in current practices. Although the critical need for transition care for AYA with rheumatic diseases is a global issue, most guidelines and recommendations are published in the West. To date, there are no publications describing rheumatology transition programs in Asia. Structured transition care is still underdeveloped in many Asian countries. A survey done in Hong Kong revealed that less than 10% of patients with chronic illnesses had ever received transition information from their pediatricians during routine visit, indicating that physicians were not well equipped to facilitate patients and their families to go through the transition process [58]. A recent Japanese survey done in 2020 showed that 57.1% of the JIA patients and 35.9% of their parents did not know what transition care is and approximately half of them did not have the opportunity to discuss transition with their rheumatologists [59].

The lack of awareness in Asia despite increased international efforts to promote transition care is concerning but cultural differences may also account for why transition care is underdeveloped in many Asian countries. Given that transition care is often influenced by local culture and healthcare systems, there are many unique aspects of transition care in Asian context which are important but not necessarily highlighted in guidelines and recommendations from Western literature. In a study published in South Korea looking at healthcare transition readiness in emerging adults with Type 1 Diabetes Mellitus (T1DM), high scores were obtained in self-perceived competency regarding tasks related to direct glucose control, such as insulin administration and glucose monitoring, when compared to dietary or exercise control [60]. This indicates that while AYA are equipped with skills related to their disease, they may not have the opportunities to establish important aspects of their own lifestyle including diet and exercise, as these aspects are typically controlled by Asian parents. Higher transition readiness scores on disease knowledge and medication management were also reported compared to communication with doctors and engagement during appointments. This could be attributed [61]. Previous studies have shown that the ability to communicate with doctors usually do not improve with age, while disease self-management skills are generally enhanced [62], implying that issues surrounding communication with healthcare providers and self-management of lifestyle may be related to cultural and social upbringing, requiring distinct intervention not apparent in current guidelines on transition care planning.

The lack of patient independence from parents was again highlighted in a Japanese national survey of adult gastroenterologists which explored their perceptions of transition care in AYA with inflammatory bowel disease (IBD) [63]. Although transfer of care to adult-oriented healthcare system was supported by 94% of the respondents, only 27% stated that they would have no hesitation in accepting patients referred from pediatric centers due to lack of patient independence as a major contributing factor. In China, parents were also less likely to foster the AYA’s independence or facilitate his/her self-management because they perceived AYA with chronic disease as more vulnerable. Therefore, they assumed more family responsibilities in caring for AYA with chronic diseases than it is in Western families [64]. In Singapore, 37% of married couples and 97% of singles aged 15 to 34 lived with their parents and were more reliant on parental influence when making decisions [65]. In the development of transition programs in Asian centers, an even greater emphasis should therefore be placed in fostering AYA independence in managing their different aspects of their life and not just disease management.

Parenting styles and family involvement in Asian families are also different from Western families [66]. While it is important to foster AYA independence, family members should not be sidelined in the transition process. Most guidelines focus on improving healthcare system processes and provision of support and knowledge only to AYA. The studies of Ersig et al., and Polfuss et al., demonstrated that caregivers’ lack of confidence in AYA’s abilities to manage their conditions and over emphasis on possible negative outcomes can lead to unsuccessful transitions and impede AYA’s self-management of chronic diseases [67,68]. It is therefore important for transition care guidelines in Asian institutions to provide family management support that focus on augmenting parents’ perception of their children and the chronic diseases. They should be convinced to promote independence and autonomy of their children during the transition process.

In Singapore, the majority of the AYA with rheumatic diseases did not report a lack of health-related knowledge as a barrier to transition but more than half of the patients are deficient in knowledge on health insurance and navigating the healthcare systems (unpublished data). This highlights that the complexity of local healthcare systems and funding is an important barrier to a successful transition and that many of the healthcare and funding systems in Asian countries are vastly different from the West. It is essential for the transition programs to approach these issues differently based on the cultural context.

## 6. Conclusions

Effective transition care for AYA is important to rheumatology care provision, especially when active disease and morbidity are still observed in adulthood in many of the rheumatic diseases. Table 1 summarizes the key elements of a transition program. Successful transition has major implications to both AYA and their families. In recent years, there has been increasing interest surrounding transition and its recognition has become more prominent in rheumatology, leading to the publication of specific guidelines. Despite all these efforts, much remain to be done. Future research will need to focus on implementing standardized outcomes measures in evaluation of transition programs and regional guidelines should also be developed with consideration of different cultural context in the Asian rheumatology population.

## Figures and Tables

**Table 1:** Key elements of a transition program.

**Key Elements of a Transition Program**
Systematic transition process with formal guidelines and policies outlining the transition process
Early preparation with emphasis on education on transition and empowerment around self-management Promoting independent communication and engagement with healthcare providers Individualized transition plan that is developmentally appropriate Introduction to adult health care system and proper transfer through shared clinic or designated transition coordinator Follow up of patients and evaluation of transition process to identify barriers and improve outcomes
